# Additive Effect of a Combination of *Artocarpus lakoocha* and *Glycyrrhiza glabra* Extracts on Tyrosinase Inhibition in Melanoma B16 Cells

**DOI:** 10.3390/ph13100310

**Published:** 2020-10-14

**Authors:** Tasanee Panichakul, Teerapat Rodboon, Prasit Suwannalert, Chanchai Tripetch, Rittipun Rungruang, Nattaporn Boohuad, Piyawan Youdee

**Affiliations:** 1 Department of Cosmetic Science, Faculty of Science and Technology, Suan Dusit University, 228-228/1-3 Sirindhorn Rd. Bangplad, Bangkok 10700, Thailand; chanchai_tre@dusit.ac.th (C.T.); rittipun_run@dusit.ac.th (R.R.); nattaporn_boo@dusit.ac.th (N.B.); 2 Department of Pathobiology, Faculty of Science, Mahidol University, 272 Rama VI Rd., Ratchathewi District, Bangkok 10400, Thailand; teerapat.rob@student.mahidol.ac.th (T.R.); prasit.suw@mahidol.ac.th (P.S.); 3 School of Culinary Arts, Suan Dusit University, 228-228/1-3 Sirindhorn Rd. Bangplad, Bangkok 10700, Thailand; piyawan_you@dusit.ac.th

**Keywords:** *Artocarpus lakoocha*, *Glycyrrhiza glabra*, oxyresveratrol, glabridin, tyrosinase, melanin

## Abstract

*Artocarpus lakoocha* (Al) and *Glycyrrhiza glabra* (Gg) extracts have been reported to show tyrosinase inhibitory activity and melanin pigment reduction. This is the first study to assess the combination of Al and Gg extracts in enhancing inhibition of tyrosinase and reduction of melanin pigments. Al and Gg extracted by maceration in 70% and 95% ethanol were analyzed for oxyresveratrol and glabridin using Ultra High Performance Liquid Chromatography. Extracts of Al and Gg singly and combinations of Al95 and Gg95 were tested for cytotoxicity, tyrosinase inhibitory activity, and reduction of melanin pigments in melanoma B16 cells. Al95 had higher antioxidant, tyrosinase inhibitory activity and reduced more melanin pigments in B16 cells compared to Al70, and exhibited higher levels of oxyresveratrol. Gg95 inhibited oxidative stress and mushroom tyrosinase better than Gg70, and exhibited higher levels of glabridin. Combinations of Al95 and Gg95 at various ratios (concentration of 0.1 mg/mL) were not cytotoxic to B16 cells. Interestingly, Al95 and Gg95 combined at a ratio 9:1 reduced melanin pigment up to 53% in B16 cells. This combination of Al95 and Gg95 extracts exhibited the additive effect of reducing melanin pigments by suppressing the expression of microphthalmia-associated transcription factor (MITF), tyrosinase (TYR) and tyrosinase-related protein-2 (TRP-2) in B16 cells. The combination of Al and Gg extracts could be developed as skin care products for hyperpigmentation treatment.

## 1. Introduction

Human skin color is primarily determined by the content of melanin, a pigment that is produced by dermal melanocytes through melanogenesis. Melanin plays an important role in photoprotection and preventing skin cancer caused by UV radiation [1]. However, hyperpigmentation disorders like melasma and actinic lichen planus are considered dermatological problems. Controlling melanogenesis is important for preventing aberrant pigmentation. Melanogenesis is regulated by tyrosinase (TYR), tyrosinase-related protein-1 and 2 (TRP-1 and TRP-2), and the microphthalmia-associated transcription factor (MITF) [2,3]. TYR is the pivotal regulator of melanin production, catalyzing the hydroxylation of tyrosine to form l-3, 4-dihydroxyphenylalanine (l-DOPA), followed by oxidation of l-DOPA to produce DOPA-quinone [4]. TRP-1 and TRP-2 function in the biosynthesis of melanin downstream of TYR. TRP-2 catalyzes the production of 5,6-dihydroxyindole-2-carboxylic acid (DHICA) from dopa-chrome, and TRP-1 oxidizes DHICA to produce indole-5,6-quinone carboxylic acid, ultimately resulting in melanin formation [5,6,7]. The tyrosinase family genes, including TYR, TRP-1, and TRP-2 are regulated by MITF [8,9,10]. MITF strongly regulates TYR gene expression and this transcription factor plays a key role in melanogenesis [5,11]. Inhibition of melanin synthesis, through disruption of melanogenesis, could improve or prevent hyperpigmentation disorders. Numerous substances have been reported as tyrosinase inhibitors and mediate their effects either as competitive and noncompetitive inhibitors [12]. Tyrosinase inhibitors include l-ascorbic acid [13,14,15], kojic acid [16,17], ellagic acid [18], tranexamic acid [19,20,21], arbutin [22], and hydroquinone [23,24]. These compounds have been proposed as skin-whitening agents for the topical treatment of hyperpigmentation disorders. However, several side effects from these skin-whitening agents have been reported. 1,4-dihydroquinone can cause reversible inhibition of cellular metabolism, which is attributed to induce melanocyte cytotoxicity and mutation [25]. In addition, kojic acid can trigger contact dermatitis [26] and has carcinogenic potential [27,28]. Therefore, there is considerable interest in finding alternative, herbal depigmenting agents.

*Artocarpus lakoocha* (Al) Roxb. (Moraceae), also known as *Artocarpus lacucha*, is a tropical tree found in South and Southeast Asia, including Southern China, India, Sri Lanka, Myanmar, Thailand, Malaysia, and Indonesia [29]. In Thailand, Ma-Haad is the common name for Al and the aqueous extract from this plant, Puag-Haad, has been traditionally used as an antihelmintic drug for treatment of tapeworm infections [30,31]. Ethanolic extracts of Al heartwood have shown potential antioxidant and tyrosinase inhibitory activities [32,33]. The active components in the Al heartwood extract with tyrosinase inhibitory activity are oxyresveratrol and resveratrol, which have potential use as skin whitening agents [31,34,35]. Hydroglycolic extracts of Al heartwood also exhibit antioxidant and in vitro tyrosinase inhibitory activities, as well as in vivo melanin-reducing effects in human volunteers. These reports indicate that Al extracts may have potential for skin-whitening agents [29,34].

*Glycyrrhiza glabra* (Gg) from the family Fabaceae, is commonly known as liquorice, is cultivated in several regions, including China, Turkey, Syria, Italy, Pakistan, and Uzbekistan [36,37]. Liquorice is widely utilized as a natural flavor extract in food and pharmaceutical products. In addition, it has been traditionally proposed for the treatment of upper and lower respiratory diseases, skin disorder, diabetes, gastrointestinal ulcers, stomach ache, and cardiovascular diseases [38]. The hydrophobic fraction from extracts of Gg contained flavonoids and exhibited inhibitory effects on melanogenesis [39,40] and extracts from Gg have been used as a depigmentation agent in cosmetic products [41]. Glabridin, a polyphenolic flavonoid compound found in Gg extract, has been reported to inhibit the tyrosinase activity of melanocytes [42] and may be the active component of Gg extracts.

Loss of effectiveness and side effects, like skin irritation, may appear when using a mono-extract as a long-term treatment. In this study, the combination of Al and Gg extracts was investigated for cellular cytotoxicity, tyrosinase inhibitory activities, and reduction of melanin contents in melanoma B16 cells. The expression of MITF, TYR, TRP-1, and TRP-2 in B16 cells was also evaluated. Enhancement of tyrosinase inhibitory activity and reduction of melanin content using a combination of Al and Gg extracts appears to be an alternative approach for treatment of hyperpigmentation disorders. This combination of Al and Gg extracts also has potential use as a whitening agent in skin care products.

## 2. Results

### 2.1. Chemical Profiles, Total Phenolic, Flavonoid Contents Antioxidant and Tyrosinase Inhibitory Activities in Extracts of A. lakoocha and G. glabra

**Figure 1:**
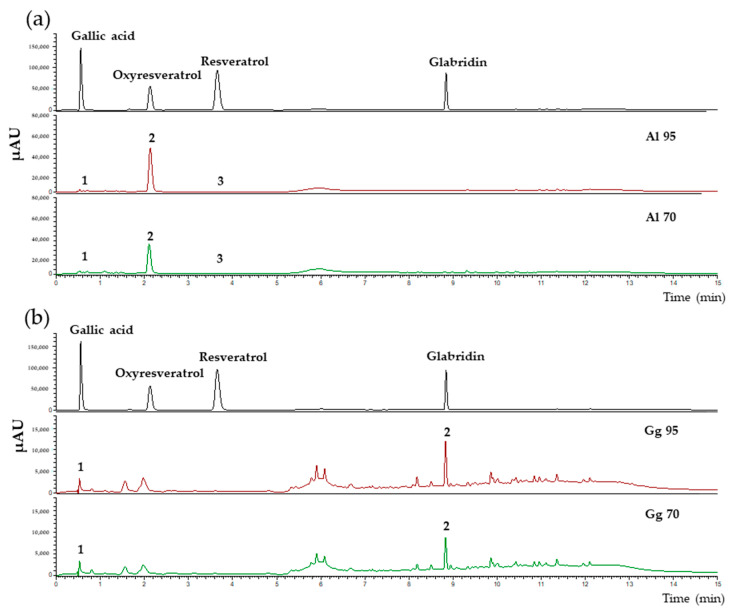
Chromatograms of *A. lakoocha* (Al) and *G. glabra* (Gg) extracts using 70% and 95% ethanol. Ultra High Performance Liquid Chromatography (UHPLC) analysis with detection at a wavelength of 280 nm. (**a**) Standards (gallic acid, oxyresveratrol, resveratrol and glabridin) and the chromatograms from Al70 and Al95 extracts show gallic acid (1), oxyresveratrol (2) and resveratrol (3); (**b**) standards (gallic acid, oxyresveratrol, resveratrol and glabridin) and the chromatograms of Gg70 and Gg95 extracts showing gallic acid (1) and glabridin (2).

The total phenolic and flavonoid contents of *A. lakoocha* (Al) and *G. glabra* (Gg) extracts prepared using either 70% or 95% ethanol (Al70, Al95, Gg70 and Gg95) were determined. Results showed that Al extracts had higher levels of total phenolic content than Gg extracts. The total phenolic content of Al95 was the highest with 398.44 ± 20.47 mg gallic acid equivalent (GAE) equivalents/g extract. The Al70, Gg70 and Gg95 extracts had 127.99 ± 5.22, 90.76 ± 11.23 and 215.07 ± 24.37 mg GAE equivalent/g, respectively. The total flavonoid contents of Al70, Al95, Gg70 and Gg95 were 2.05 ± 0.09, 8.79 ± 0.40, 5.87 ± 0.56 and 9.20 ± 0.84 mg quercetin equivalent (QE) equivalent/g extract, respectively. This analysis indicated that the flavonoid contents from the Gg extracts were higher than those in the Al extracts. Analysis of Al70, Al95, Gg70 and Gg95 extracts by Ultra High Performance Liquid Chromatography (UHPLC) revealed the presence of several chemical constituents (Figure 1). The chemical compounds in Al70 and Al95 extracts included gallic acid (0.56 min), oxyresveratrol (2.12 min) and resveratrol (3.75 min). One prominent peak for oxyresveratrol was found in both Al70 and Al95 extracts (Figure 1a). The concentrations of oxyresveratrol were 113.10 ± 0.48 and 149.58 ± 0.43 mg/g of extract for Al70 and Al95 extracts, respectively. Gallic acid and resveratrol were also found at low concentrations in Al70 and Al95 extracts. As shown in Figure 1b, major chemical compounds in Gg70 and Gg95 extracts included gallic acid (0.56 min) and glabridin (8.84 min). The concentrations of glabridin were 5.56 ± 0.11 and 12.48 ± 0.48 mg/g of extract from Gg70 and Gg95 extracts, respectively. Glabridin was the major peak found in both Gg70 and Gg95 with gallic acid present at low levels in Gg extracts.

The possible inhibitory effects of Al and Gg extracts on mushroom tyrosinase were analyzed. The results are presented as the concentration that inhibited 50% of the mushroom tyrosinase activity (IC_50_) (Table 1). Al70, Al95, Gg70 and Gg95 extracts inhibited mushroom tyrosinase activity with IC_50_ values of 0.028, 0.017, 0.114 and 0.074 mg/mL, respectively. These values indicate that the Al and Gg extracts were more potent than kojic acid (IC_50_ = 0.495 mg/mL) as tyrosinase inhibitors. Al had higher antioxidant and tyrosinase inhibitory activities than those of Gg extracts in Table 1. Al95 exhibited the highest inhibitory effect on tyrosinase and this correlated with the total phenolic content and antioxidant activity of this extract. These results suggest that Al and Gg possessed anti-melanogenesis activity with a rank order of efficacy for Al and Gg to inhibit mushroom tyrosinase activity of Al95 > Al70 > Gg95 > Gg70. The main chemical compounds found in crude extracts of *A. lakoocha* and *G. glabra* were oxyresveratrol and glabrindin, respectively.

### 2.2. Cytotoxicity of A. lakoocha and G. glabra Extracts in B16 Cells

**Figure 2:**
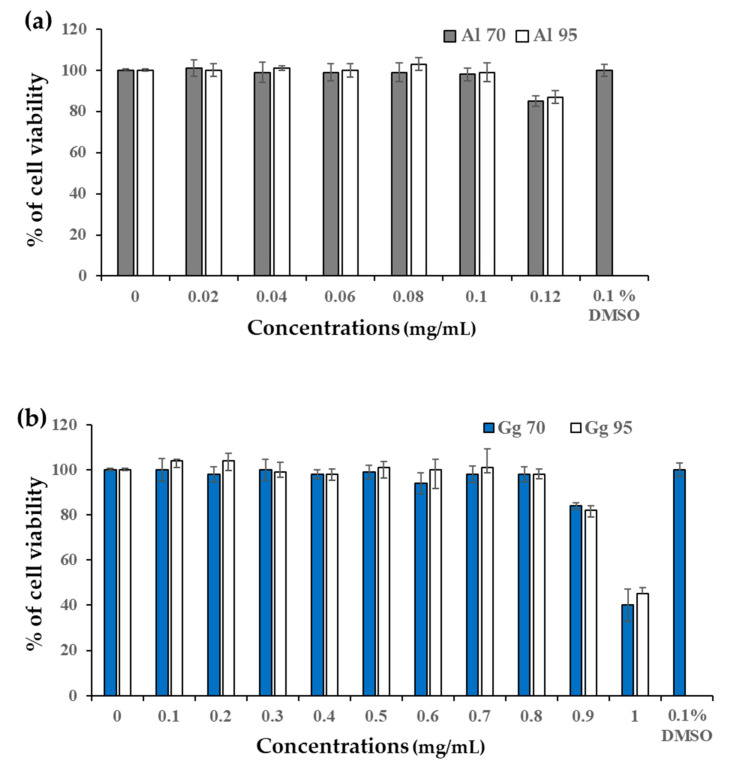
Viability of B16 cells exposed to *A. lakoocha* and *G. glabra* extracts. (**a**) *A. lakoocha* macerated in 70% (Al70) and 95% (Al95) of ethanol; and (**b**) *G. glabra* macerated in 70% (Gg70) and 95% (Gg95) of ethanol. Percentages of cell viability means ± SD from three independent experiments.

Cell viability of all extracts was analyzed using the 3-(4,5-dimethylthaizol-2-yl)-2,5-diphenyltetrazolium bromide (MTT) assay to establish non-cytotoxic concentrations of the extracts from *A. lakoocha* and *G. glabra* (Figure 2). Survival with more than 95% of the B16 cells was used to determine the safety concentration of *A. lakoocha* and *G. glabra* extracts. The concentration of extracts that resulted in a cell viability of 98–99% was 0.1 mg/mL for Al70 and Al95 (Figure 2a) and 0.8 mg/mL for Gg70 and Gg95 (Figure 2b). These concentrations of Al and Gg extracts were considered safe and selected as the highest dose for use in analysis of their effects on cellular melanin content and tyrosinase activity.

### 2.3. A. lakoocha and G. glabra Extracts Decreased Cellular Melanin Content and Tyrosinase Activity

**Figure 3:**
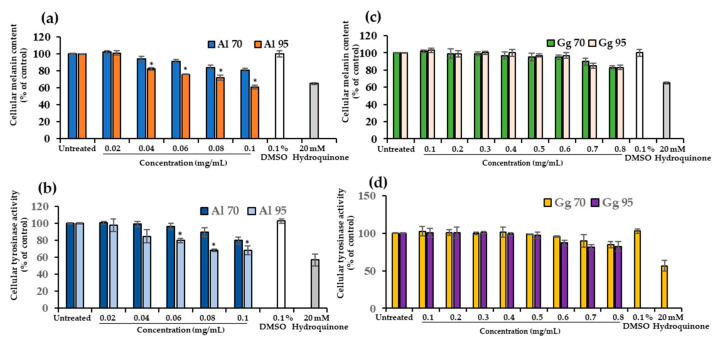
Effect of *A. lakoocha* and *G. glabra* extracts on cellular melanin content and tyrosinase activity. B16 cells were treated with Al70 and Al95 and evaluated for (**a**) cellular melanin content and (**b**) cellular tyrosinase activity. B16 cells treated with Gg70 and Gg95 monitored for (**c**) cellular melanin content and (**d**) cellular tyrosinase activity. Cells were treated with extracts for 48 h and data are means ± SD from three independent experiments. The superscript letters (*) indicate significant difference at *p* < 0.01.

The inhibitory effect of Al and Gg extracts on cellular melanin content and tyrosinase activity in B16 cells is illustrated in Figure 3. B16 cells were treated with non-cytotoxic concentrations of Al (0.02–0.1 mg/mL) and Gg (0.1–0.8 mg/mL). Al and Gg produced a dose-dependent decrease in the melanin content (Figure 3a,c). The inhibitory effects of Al95 on melanin synthesis and tyrosinase activity were significantly higher than those of Al70 (*p* < 0.01). This difference was noted for at Al concentrations ranging from 0.06 to 0.1 mg/mL. Al95 at a concentration of 0.1 mg/mL was able to reduce melanin content 39% (Figure 3a) and tyrosinase activity by 34% (Figure 3b). In contrast, Gg70 and Gg95 extracts, at concentrations of 0.7 and 0.8 mg/mL, decrease melanin content by 10% to 17% (Figure 3c) and tyrosinase activity by 10% to 19% (Figure 3d). No significant differences were found in the effects of Gg70 and Gg95 (*p* < 0.01) extracts. These results indicate that Al reduced the cellular accumulation of melanin pigment by inhibiting tyrosinase activity. However, a higher concentration of Gg resulted in a more limited effect on inhibition of tyrosinase activity as well as cellular content of melanin pigment.

### 2.4. Combined Effects of A. lakoocha and G. glabra Extracts on Cellular Melanin Content and Tyrosinase Activity

**Figure 4:**
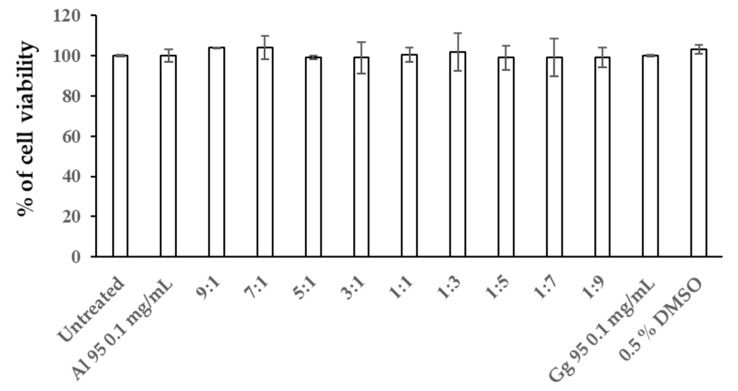
Cell viability of B16 cells exposed to mixtures of *A. lakoocha* and *G. glabra* extracts. Cells were treated with the combination of Al95 and Gg95 extracts for 72 h. Percentages of cell viability are means ± SD from three independent experiments.

**Figure 5:**
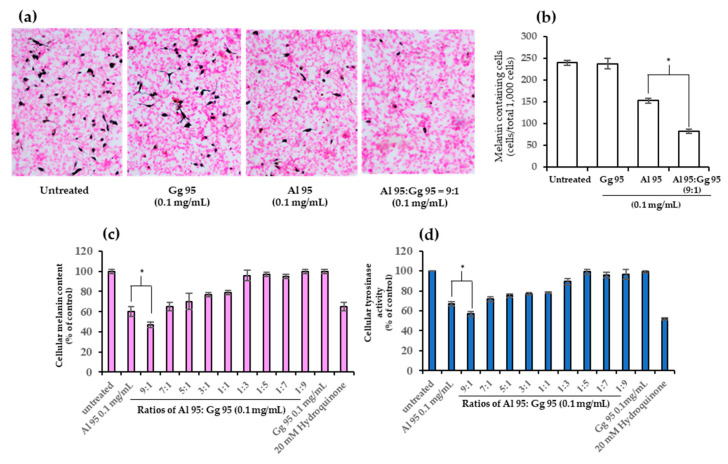
Effect of combined *A. lakoocha* and *G. glabra* on cellular melanin content and tyrosinase activity. (**a**) Melanin pigments (black) in B16 cells (pink) observed under a light microscope (Magnification ×100); (**b**) the number of melanin containing cells from a total of 1000 cells counted under a light microscope; (**c**) cellular melanin content; (**d**) cellular tyrosinase activity. Cells were treated with combinations of Al95 and Gg95 at a final concentration of 0.1 mg/mL in several ratios for 48 h. The data are means ± SD from three independent experiments. The superscript letters (*) indicate significant difference at *p* < 0.01.

To determine the combined effects in B16 cells from *A. lakoocha* and *G. glabra* extracts on cytotoxicity, cellular melanin content, and tyrosinase activity, Al95 and Gg95 extracts were used in combination, as shown in Figure 4 and Figure 5. Cells were treated with Al95 and Gg95 extracts, alone and in combination with Al95 and Gg95 at the following ratios: 9:1, 7:1, 5:1, 3:1, 1:1, 1:3, 1:5, 1:7, and 1:9. The viability of B16 cells treated with Al and Gg extract at all ratios was nearly 100% of untreated control cells after 72 h (Figure 4). The combination of Al95 and Gg95 extracts did not increase cytotoxicity toward B16 cells and no cytotoxicity was observed at a concentration of 0.1 mg/mL. Interestingly, the combined Al95 and Gg95 extracts were able to decrease melanin content in B16 cells (Figure 5a–c). Al95 and Gg95 extracts combined at a ratio of 9:1 was especially effective in reducing the number of melanin containing cells (82 ± 4.64 cells/total 1000 cells) (Figure 5b) and melanin content, showing a reduction of up to 53% (Figure 5c). This was a significant reduction (*p* < 0.01) compared to Al95 extract alone, which exhibited the number of melanin containing cells 153 ± 5.79 cells per total 1000 cells and only 35% reduction in melanin pigment. Tyrosinase inhibitory activity was also significantly enhanced with this combined extract in B16 cells (*p* < 0.01) compared to Al95 alone (Figure 5d). These results indicate that the combination of *A. lakoocha* and *G. glabra* extracts has an additive effect to inhibit tyrosinase activity, leading to a reduction in cellular melanin pigments without causing cytotoxicity.

### 2.5. Effect of Combined A. lakoocha and G. glabra Extracts on Proteins Related to Melanogenesis in B16 Cells

**Figure 6:**
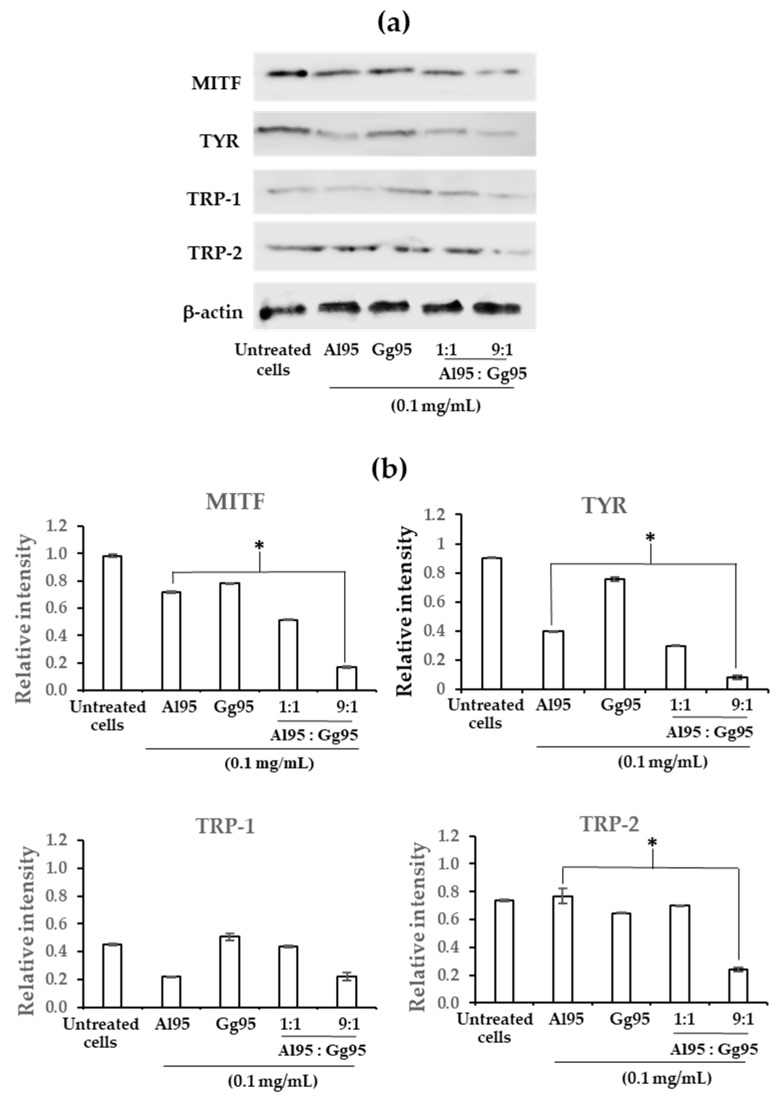
Inhibitory effect of combined *A. lakoocha* and *G. glabra* extracts on proteins related to melanogenesis. (**a**) Protein bands for microphthalmia-associated transcription factor (MITF), tyrosinase (TYR), tyrosinase-related protein (TRP)-1, TRP-2 and β-actin. (**b**) Relative intensity of protein levels of MITF, TYR, TRP-1, and TRP-2. Cells were treated with mixtures of Al95 and Gg95 at final concentration of 0.1 mg/mL at ratios of 1:1 and 9:1 for 48 h. The samples derive from the same experiment and blots were processed in parallel. The data are means ± SD from three independent experiments. The superscript letters (*) indicate significant difference at *p* < 0.01.

To identify a potential mechanism leading to reduced melanin accumulation following exposure to Al95 and Gg95 extracts, expression levels of melanogenesis-related proteins, including MITF, TYR, TRP-1 and TRP-2 were evaluated by Western blots (Figure 6a). Combination of Al95 and Gg95 extracts at a ratio of 9:1 significantly reduced MITF, TYR, and TRP-2 protein levels (*p* < 0.01) compared with Al95 alone (Figure 6b). The Al95 and Gg95 combination resulted in the maximum reduction of melanogenesis proteins among all conditions examined. The present findings suggest that the combination of Al95 and Gg95 extracts had an additive effect on the down regulation of MITF, TYR, and TRP-2 expression. The hypopigmentation effect due to exposure to the combination of Al95 and Gg95 extracts is likely mediated through down-regulation of MITF gene expression, which would subsequently repress the expression of tyrosinase and TRP-2.

## 3. Discussion

In this study, a combination of Al and Gg extracts was investigated for cellular tyrosinase inhibitory activity, reduction of melanin contents and downregulated expression of melanogenesis-related genes MITF, TYR, TRP-1 and TRP-2 in melanoma B16 cells. This is the first report exhibiting additive activity of the combination of Al and Gg extracts to inhibit tyrosinase and reduce melanin pigments by suppressing the expression of MITF, TYR and TRP-2 in B16 cells.

Previously, *Artocarpus lakoocha* (Al) extracted with 95% ethanol was shown to contain higher total phenolic content and exhibit higher tyrosinase inhibitory activity compared to Al extracted with propylene glycol [29]. A high total phenolic content was also reported for Al extracted with 80% ethanol [33]. Extraction of Al using water, methanol, ethanol, propanol and butanol demonstrated that ethanol was the most effective solvent for isolating oxyresveratrol and resveratrol [43,44]. Similarly, *Glycyrrhiza glabra* (Gg) extracted with ethanol had higher total phenolic and flavonoid contents than water extraction [45]. Glabribin and glycyrrhizic were more abundant from Gg extracted in ethanol compared to extracted that with water, methanol, acetonitrile, and chloroform [46]. In this study, Al and Gg were macerated in 70% and 95% ethanol and higher antioxidant and tyrosinase inhibitory activity was found when using 95% ethanol. These results were consistent with the levels of total phenolic and flavonoid contents found in Al95 and Gg95 extracts. In particular, oxyresveratrol and glabridin were more abundant in Al95 and Gg95 extracts, respectively, compared to extracts prepared using 70% ethanol. Al and Gg extracted with ethanol are expected to contain oxyresveratrol and glabridin, respectively, and these compounds appear to possess tyrosinase inhibitory activity. The mushroom tyrosinase test is a simple analytical method to assess inhibitory activity of compounds or extracts. However, mushroom tyrosinase is different from mammalian tyrosinase due to its unique requirements for substrate and cofactors. In addition, the sensitivity of mushroom tyrosinase to inhibitors is distinct compared to the mammalian enzyme [47]. Another difference arises from protein modifications to these tyrosinase proteins. Mushroom tyrosinase is secreted, while mammalian tyrosinase is present in an inactive form that requires glycosylation in the Golgi apparatus and phosphoactivation by protein kinase C beta (PKCβ) [48,49,50]. Many plant extracts that show in vitro inhibitory activity against mushroom tyrosinase are unable to reduce pigmentation in cells [51]. Previously, crude extracts isolated from *A. lakoocha* and *G. glabra* have been found to have tyrosinase inhibitory activities and the ability to reduce melanin pigmentation as well as containing antioxidant and antimicrobial activities [34,41,42,43,45,52,53]. Similarly, Al and Gg extracts in this study were able to inhibit both mushroom and cellular tyrosinase activities and reduce melanin pigments in B16 cells.

Mixtures of compounds can often lead to more pronounced effects than the individual products [54,55]. Inhibition of melanin production by a combination of plant extracts had been reported previously. A combination of Siberian larch and pomegranate extracts exhibited a two-fold reduction in melanin content compared to S. larch or pomegranate extracts alone with no cytotoxicity to the cell. These combined extracts reduced expression of melanocyte specific genes, TYR, MITF, and melanosome structural proteins (melanocyte-specific glycoprotein (Pmel17) and melanoma antigen recognized by T cells 1 (Mart1)); however, inhibition of the tyrosinase enzyme was not observed [56]. A combination of chia seed and pomegranate fruit extracts exhibited an additive effect on the inhibition of melanin biosynthesis also with no corresponding effect on tyrosinase activity. The combination of chia seed and pomegranate fruit extracts is likely due to decreased expression of melanogenesis-related genes (TYR, TRP1, and melanocortin 1 receptor (MC1R)) [57]. In our study, a combination of Al95 and Gg95 extracts, at concentrations that did not reduce the viability of B16 cells, was able to decrease melanin pigments as well as inhibit tyrosinase activity. These results indicate that a combination between Al95 and Gg95 extracts additively effect the reduction of cellular melanin pigments. Investigation into the possible mechanism of action revealed that Al95 extract alone downregulated expression of melanogenesis-related genes MITF, TYR, and TRP-1. While a combination of Al95 with Gg95 extracts more significantly reduced expression of MITF, TYR, and TRP-2. The hypopigmentation effect of a combination of Al95 and Gg95 extracts may potentially be the result of down-regulation of MITF gene expression. Reduced MITF levels may affect in turn repress the expression of tyrosinase and TRP-2, as MITF is a major regulator of the synthesis of tyrosinase-related proteins (TRPs), including TYR, TRP-1 and TRP2 [58,59].

Numerous topical products for skin whitening are available and contain active ingredients to reduce melanin production. Ascorbic acid and its derivatives, as active ingredients with anti-tyrosinase properties have been used in some commercial lightening products [60]. Ascorbic acid acts as a reducer to block the chain of oxidations transforming tyrosine into melanin and interacts with copper, an essential cofactor in tyrosinase activity [61]. This active ingredient is not used alone but always used in combination with another ingredient. *A. lakoocha* extract was reported to exhibit in vitro tyrosinase inhibitory activity as well as the ability to reduce in vivo melanin content in a human volunteer. It was suggested based on these findings that *A. lakoocha* extract has been proposed as a potential skin-whitening agent [34,62]. However, another report raised the concern that at high concentrations the *A. lakoocha* ethanolic extract may be toxic in the blood system [33]. Glabridin and glabrene from *G. glabra* are known to inhibit tyrosinase activity and have been used as depigmentation agents in cosmetics [25,42]. Induction of melanin pigments by UV-B was reduced by topical application of 0.5% glabridin [42]. The main drawbacks of glabridin are its poor skin-penetrating ability and instability in formulations [25]. The combination of Al95 and Gg95 extracts has an additive effect on the reduction of melanin pigments, an alternative way to reduce the cytotoxicity of compounds in Al95 extracts and improve skin-penetration of compound Gg95 extracts; however, this will be further investigated. Finally, this is the first study suggesting that combination between *A. lakoocha* and *G. glabra* extracts is useful in development of skin care products for skin hyperpigmentation disorders.

## 4. Materials and Methods

### 4.1. Chemicals

Tyrosinase from mushroom (SLBJ5647, activity of 5771 unit/mg), 3,4-dihydroxy-l-phenylalanine (l-DOPA), dimethyl sulfoxide (DMSO), Folin and Ciocalteu’s Phenol reagent, 2,2-diphenyl-l-picrylhydrazyl (DPPH), sodium dihydrogen phosphate, 3-(4,5-dimethylthaizol-2-yl)-2,5-diphenyltetrazolium bromide (MTT), kojic acid, gallic acid, quercetin, oxyresveratrol, resveratrol, and glabridin were purchased from Sigma-Aldrich, Inc. (St. Louis, MO, USA). Disodium hydrogen phosphate and disodium carbonate were supplied by Merck Millipore (Temecula, California, USA). l-ascorbic acid and aluminium choride were purchased from Ajax Finechem (Taren Point, Australia).

### 4.2. Plant Materials and Extraction

Dried powder of *A. lakoocha* (Al) heartwood and *G. glabra* (Gg) root was purchased from Vejpongosot Pharmacy (Bangkok, Thailand). The extraction methods of Al and Gg were modified from those previously described [43]. Briefly, one kilogram of dried plant material was macerated overnight in 5 L of 70% or 95% ethanol at room temperature for Al and at 48 °C for Gg. After three rounds of maceration, the alcoholic extracts were pooled, filtered, and evaporated under reduced pressure below 45 °C. The yields of crude extracts for Al70, Al95, Gg70 and Gg95 were 9.703%, 8.675%, 8.46% and 8.153%, respectively.

### 4.3. Determination of Total Phenolic Content

The total phenolic compounds in Al and Gg extracts were detected using the Folin–Ciocalteu reagent, [63]. In 96-well plates, 4.5 μL of 1 mg/mL extracts was diluted in 126 μL of deionize water, the samples were mixed with 90 μL of 2% Na2CO3 for 3 min and then 4.5 μL of 50% Folin–Ciocalteu reagent was added. The samples were incubated at room temperature for 30 min and the resulting blue molybdenum–tungsten complex was monitored at 750 nm using a microplate reader (Biochrom EZ Read 2000, Cambridge, UK). The total phenolic content in each sample was calculated by comparison to a gallic acid standard and results are presented as milligrams of gallic acid equivalent to one gram of extract (mg GAE/g extract).

### 4.4. Determination of Flavonoid Content

The flavonoid compounds of Al and Gg extracts were determined using the aluminum chloride assay [64]. In 96-well plates, 100 μL of 1 mg/mL extracts were mixed with 100 μL of 2% AlCl3. Following incubation at room temperature for 10 min, the absorbance was monitored at 415 nm with a microplate reader (Biochrom EZ Read 2000, Cambridge, UK). The total flavonoid content of samples was analyzed by comparison to quercetin standard and the results were presented as quercetin equivalent (mg QE/g extract).

### 4.5. Phytochemical Screening Assay

Chemical composition was determined using Ultra High Performance Liquid Chromatography (UHPLC) modified as previously described [31,43]. UHPLC analysis was performed using a Thermo Scientific UHPLC UltiMate 3000 (Waltham, MA, USA) with a Hypersil GOLD™ a Q column (100 × 2.1 mm i.d., 1.9 µm, Thermo Scientific™) and results were analyzed with Thermo Scientific™ LCQUAN™ software. Crude extracts of Al and Gg and standard solutions (oxyresveratrol, resveratrol, gallic acid and glabridin) were dissolved in methanol at concentrations of 1 and 10 mg/mL. Samples were filtered (0.20 mm, Millipore) and 1 μL was directly injected. Solvents for HPLC analysis were formic acid (0.1% *v*/*v*) in water as solvent A and formic acid (0.1% *v*/*v*) in methanol as solvent B, at a flow rate of 0.5 mL/min. These experiments used the following gradient: 30% B linear (0–4 min), 30–50% B linear (4–5 min), 50–70% B linear (5–8 min), 70–100% B linear (8–12 min), 100% B (12–15 min), and 30% B linear (15–18 min). An equilibrium period of 5 min was performed prior to the injection of subsequent samples. Chromatograms were recorded at 280 nm (glabridin and gallic acid), 305 nm (resveratrol), and 326 nm (oxyresveratrol), using the photodiode detector. Quantitative determination of compounds was performed using peak area with an external standard.

### 4.6. 2,2-di-phenyl-1-picrylhydrazyl (DPPH) Radical Scavenging Assay

Antioxidant activity of Al and Gg extracts was determined based on DPPH scavenging ability [65]. Seventy-five microliters of the extracts at various concentrations (0.015–1 mg/mL) were mixed with 150 μL of 0.2 mM DPPH, incubated for 30 min, and the absorbance at 515 nm was monitored using a microplate reader (Biochrom EZ Read 2000, UK). Ethanol was used as the blank solution and L-ascorbic acid was the positive control. The scavenging activity of DPPH radicals was calculated and expressed as a percentage of the blank. The IC_50_ value represents the concentration of extract capable of reducing DPPH by 50%, calculated using a linear regression graph.

### 4.7. Mushroom Tyrosinase Assays

Inhibition of mushroom tyrosinase activity was detected using L-3,4-dihydroxyphenylalanine (l-DOPA) as a substrate [66]. Briefly, 20 μL of extracts at various concentrations (0.0024–1.25 mg/mL), 140 μL of 20 mM phosphate buffer (pH 6.8), and 20 μL of 461.68 unit/mL of mushroom tyrosinase were added to each well of a 96-well plate. Samples were mixed for 10 min, 20 μL of 4 mM l-DOPA was then added and this was followed by incubation at 37 °C for 30 min. The relative amount of dopachrome formed in the mixture was monitored by measuring the absorbance at 475 nm using a microplate reader (Biochrom EZ Read 2000, UK). Tyrosinase and l-DOPA only solutions were used as a negative control. Kojic acid at concentrations ranging from 0.0024 to 1.25 mg/mL was a positive control. The inhibition of tyrosinase activity was calculated and presented as a percentage of the control. The IC50 for tyrosinase inhibition was calculated using a linear regression graph and is the concentration of extract capable of reducing tyrosinase activity to 50%.

### 4.8. Cell Viability Assay

Cell viability of B16 cells was determined with the MTT assay to establish the non-cytotoxic concentrations of Al and Gg extracts [67]. B16 melanoma cells (RIKEN Cell Bank, Tsukuba, Japan) were cultured in Dulbecco’s Modified Eagle’s Medium (DMEM, Gibco^®^ /Thermo Scientific, Waltham, MA, USA) with 10% fetal bovine serum (FBS) and penicillin/streptomycin (50 μg/mL) in an incubator with 5% CO2 at 37 °C. Briefly, 3.0 × 10^4^ cells were plated into 96-well plates and incubated at 37 °C overnight. After removal of the culture medium, cells were treated for 72 h wth 200 μL of extracts, diluted with medium at various concentrations of 0.02–0.12 mg/mL and 0.1–1 mg/mL for the Al and Gg extracts, respectively. The culture media with extracts was removed and treated cells were then added with 100 μL of 0.5 mg/mL MTT solution. After additional 2 h, the formazan crystals were dissolved with 100 μL of dimethyl sulfoxide (DMSO). The absorbance was monitored at 530 nm using a microplate reader (1420 Victor 2, Wallac, Ramsey, MN, USA). The results were presented as the percentage of viable cells (% cell viability) by comparing with untreated cells as a control.

### 4.9. Melanin Content Assay

Melanin content in B16 cells was evaluated as described previously [68]. In brief, 1.5 × 10^5^ B16 cells were plated into 24-well plates and incubated overnight to allow cells to adhere. Cells were treated for 48 h with various concentrations of 0.02–0.1 mg/mL and 0.1–0.8 mg/mL for Al and Gg extracts, respectively. After cells were harvested using trypsin, the cell pellet was resuspended with 100 μL of 1 N NaOH, 1% Triton X-100, 1 mM phenylmethanesulfonyl fluoride (PMSF) and incubated at 60 °C for 1 h to allow cell lysis. Melanin content of lysates was monitored at 405 nm using a microplate reader (1420 Victor 2, Wallac, USA). Hydroquinone at 20 mM was used as a positive melanin inhibitor. Melanin content was presented as a percentage of the value obtained for untreated control cells.

Accumulation of melanin pigments and cellular morphology of B16 cells were observed with a Masson-Fontana stain using a procedure modified from that previously described [69]. In brief, 5.0 × 10^5^ B16 cells/mL were plated into 6-well plates for 24 h. Cells were treated with non-toxic doses of extracts and incubated for 3 days. Treated cells were fixed with absolute ethanol and rehydrated with distilled water. Cells were then stained with an ammoniacal silver solution for 24 h, sodium thiosulfate for 5 min and Mayer’s Carmalum for 10 min. Melanin pigments and cellular morphology were observed under a light microscope at a magnification of 100×. The number of melanin containing cells were calculated from a total of 1000 cells counted.

### 4.10. Cellular Tyrosinase Activity Assay

Tyrosinase activity from B16 cells was determined using l-DOPA [70]. B16 cells, 1.0 × 10^5^ cell/well were plated into a 24-well plate and incubated overnight to allow cells to adhere. Cells were treated for 48 h with various concentrations of 0.02–0.1 mg/mL and 0.1–0.8 mg/mL for Al and Gg extracts, respectively, and harvested. Cell pellets were lysed with 100 µL of 0.1% Triton X-100 and 0.1 mM PMSF (pH 7.5) in phosphate buffer saline (PBS). Lysates were clarified by centrifugation at 12,000× *g* at 4 °C for 15 min to obtain the supernatant. Reaction mixtures consisting of 80 μL of supernatant and 20 μL of 20 mM l-DOPA were determined after incubation at 37 °C for 60 min. The optical densities were measured at 492 nm using a microplate reader (1420 Victor 2, Wallac, Ramsey, MN, USA). Sodium l-lactate (SLL) at a concentration of 20 mM was used as a positive control. The cellular tyrosinase activity was calculated and presented as a percentage of the control (100% for untreated cells).

### 4.11. Evaluation of Additive Effects of Al and Gg Extracts on Cellular Melanin Content and Tyrosinase Activity

Combinations between Al95 and Gg95 extracts were evaluated for inhibition of tyrosinase activity and melanin production in B16 cells. Mixtures of Al and Gg extracts was prepared at ratios of 9:1, 7:1, 5:1, 3:1, 1:1, 1:3, 1:5, 1:7 and 1:9. The final concentration of all combined extracts of Al and Gg was 0.1 mg/mL. These combined extracts were evaluated for cytotoxicity, inhibition of tyrosinase activity, and effects on melanin pigments in melanoma B16 cells.

### 4.12. Western Blot Analysis

The abundance of proteins involved in melanogenesis was determined using Western blot analysis, modified from the procedure previously described [71]. Briefly, 1.0 × 10^5^ cells were plated into the 6 well plates and incubated at 37 °C, 5% CO2 for 24 h. Following removal of the old media, 2 mL of conditioned media containing combined Al and Gg extracts at ratios of 1:1 and 9:1 and single extract in final concentration at 0.1 mg/mL was added and samples were incubated at 37 °C, 5% CO2 for 48 h. Treated cells were harvested and lysed in 100 μL radioimmunoprecipitation assay buffer (RIPA) buffer. Protein content was determined using a protein quantitation kit (Merck, Darmstadt, Germany). Total protein, 20 μg, from each sample was separated using 12% SDS-PAGE and transferred to polyvinylidene difluoride (PVDF) membranes. Membranes were treated separately with the following primary antibodies at the indicated dilutions at room temperature for 1 h with gentle agitation: rabbit anti-mouse tyrosinase (immunoglobulin G (IgG) (1:100), rabbit anti-mouse TRP-1 IgG (1:200), rabbit anti-mouse TRP-2 IgG (1:100), goat anti-mouse MITF IgG (1:200) and rabbit anti-mouse β-actin IgG (1:500). The membranes were treated with secondary anti-mouse or goat IgG conjugated with horseradish peroxides (1:2000). Protein bands were visualized with enhanced chemiluminescence (ECL) with a Gel documentation system and quantitated using Image J software.

### 4.13. Statistical Analysis

All grouped data were evaluated with SPSS version 17. Data were expressed as the mean ± SD of three independent experiments and analyzed for statistical significance using t-test and one-way ANOVA. Differences were considered statistically significant at *p* < 0.01.

## 5. Conclusions

*Artocarpus lakoocha* (Al) and *Glycyrrhiza glabra* (Gg) extracts prepared using 95% ethanol had high antioxidant and tyrosinase inhibitory activity as well as a high total phenolic and flavonoid contents. Oxyresveratrol and glabridin were major components found in Al95 and Gg 95 extracts, respectively, and these compounds appear to possess tyrosinase inhibitory activity. Interestingly, Al and Gg extracts exhibited the inhibition of both mushroom and cellular tyrosinase activities, and reduction of melanin pigments in B16 cells. A combination of Al95 and Gg95 extracts was able to reduce melanin pigments and enhance tyrosinase inhibitory activity in B16 cells when compared to Al95 alone, without decreasing cell viability. The combination of Al95 and Gg95 extracts appears to exhibit an additive effect to reduce melanin pigments. The enhanced inhibitory effect of the Al and Gg extract mixture may be due to the action of oxyresveratrol and glabridin. Based on the findings, we propose that an effective melanin reduction strategy should target multiple pathways of melanin synthesis through the action of a mixture of ingredients. These findings suggest that the combination of *A. lakoocha* and *G. glabra* extracts is a new approach to treat skin hyperpigmentation disorders.

## Figures and Tables

**Table 1:** Total phenolic and flavonoid content, antioxidant activity, and mushroom tyrosinase inhibitory activity of *A. lakoocha* (Al) and *G. glabra* (Gg).

Extracts	Tyrosinase Inhibitory Activity IC_50_ (Mean ± SD) (mg/mL)	Radical Scavenging Activity IC_50_ (Mean ± SD) (mg/mL)
Al70	0.028 ± 0.0029	0.081 ± 0.027
Al95	0.017 ± 0.0024 ^1^	0.074 ± 0.024
Gg70	0.114 ± 0.0057	0.365 ± 0.171
Gg95	0.074 ± 0.0011	0.223 ± 0.072
Kojic acid	0.495 ± 0.029	-
Ascorbic acid	-	0.007 ± 0.0008

Data are means ± SD from three independent experiments. (^1^) indicate significant difference at *p* < 0.01. IC_50_, the concentration of samples that inhibited 50% of tyrosinase inhibitory or antioxidant activities.
